# A multi-scale review of the dynamics of collective behaviour: from rapid responses to ontogeny and evolution

**DOI:** 10.1098/rstb.2022.0059

**Published:** 2023-04-10

**Authors:** Christos C. Ioannou, Kate L. Laskowski

**Affiliations:** ^1^ School of Biological Sciences, University of Bristol, Bristol, BS81TQ, UK; ^2^ Department of Evolution and Ecology, University of California Davis, Davis, CA 95616, USA

**Keywords:** collective motion, division of labour, group decision making, leadership, development, timescale

## Abstract

Collective behaviours, such as flocking in birds or decision making by bee colonies, are some of the most intriguing behavioural phenomena in the animal kingdom. The study of collective behaviour focuses on the interactions between individuals within groups, which typically occur over close ranges and short timescales, and how these interactions drive larger scale properties such as group size, information transfer within groups and group-level decision making. To date, however, most studies have focused on snapshots, typically studying collective behaviour over short timescales up to minutes or hours. However, being a biological trait, much longer timescales are important in animal collective behaviour, particularly how individuals change over their lifetime (the domain of developmental biology) and how individuals change from one generation to the next (the domain of evolutionary biology). Here, we give an overview of collective behaviour across timescales from the short to the long, illustrating how a full understanding of this behaviour in animals requires much more research attention on its developmental and evolutionary biology. Our review forms the prologue of this special issue, which addresses and pushes forward understanding the development and evolution of collective behaviour, encouraging a new direction for collective behaviour research.

This article is part of a discussion meeting issue ‘Collective behaviour through time’.

## 1. Introduction

The benefits and costs of living in groups have been studied extensively [1–3], and variation in these benefits and costs helps explain why there is such diversity in social behaviour in the animal kingdom. Avoidance of predation risk is a widespread explanation for group living, while competition for food and disease transmission are common costs. While the behavioural ecology of living in groups has a long history of research, more recently attention has shifted to what is known as collective behaviour: the mechanisms that allow animals to maintain groups and transfer information, and how interactions between individuals give rise to group-level properties such as shape and spatial positioning within groups, group movement speed and direction, information transfer within groups, and collective decision making. These group-level properties are often emergent, as they are difficult to predict from the simple inter-individual interactions that drive them, which lack centralized or external control and are hence self-organized [4–6].

The mechanisms underlying collective behaviour are typically local-scale, i.e. involve interactions between individuals physically close to one another in the group, and occur over short timescales. For example, the response of a fish to the sudden change of direction of a near neighbour can occur in a fraction of a second, resulting in coordinated movement of the entire school [7]. Understandably, the field of collective behaviour has thus focused on these short timescales. However, longer timescales are important for all biological traits, including how they change within the lifetime of an individual (ontogeny) and over evolutionary time. Niko Tinbergen [8], recipient of the 1973 Nobel Prize for co-founding the modern study of animal behaviour, emphasized that a full understanding of a behavioural trait requires an understanding of these developmental and evolutionary histories. The other two questions of Tinbergen's ‘Four Questions’, the functional adaptive reason(s) why a behaviour occurs and the proximate mechanism(s) of how it occurs, are now relatively well researched for collective behaviours. While developmental and evolutionary changes are pillars of modern biology, their importance in collective behaviour has been mostly neglected [9]. Thus, this special issue ‘Collective animal behaviour through time’ aims to encourage research in the developmental and evolutionary aspects of collective behaviour.

There is an incredible diversity of collective behaviours in a wide range of animals that have now been studied, which can make it difficult to clearly define a threshold timescale that separates the relatively well-studied ‘short’ timescales compared to longer timescales. Given the clear importance of collective behaviour to the biology of social insects, it is not surprising that the study of collective behaviour developed most rapidly in these animals [4,10], with some research attention being given, independently of the research in social insects, to shoaling in fishes [6,11]. With technological advancements making the computer simulation of multiple individuals interacting simultaneously feasible (i.e. agent-based models [12,13]) and the application of the complex systems approach from the physical sciences [14], the similarities in collective behaviour across diverse taxonomic groups have become evident and allowed general principles to become established across taxa [5]. Advances in technology to obtain empirical data from a wider range of animal groups, particularly under natural conditions [15], have now allowed the collective behaviour of birds and mammals to also be studied more extensively [16–21]. This taxonomic diversity is a strength of this research area, although it does raise a question of how we define timescales: while a shoal of fish can make a decision regarding which of two arms of a maze to swim into within seconds [22], a seemingly similar binary decision can take hours in an ant colony and involve multiple phases [23,24]. Investigations of collective decision making can even extend into multiple days, which we still consider to be a collective behaviour that occurs over a short timescale; nest-site selection in honeybees (*Apis mellifera*) is a particularly well-researched example [25]. Rather than distinguishing short versus long timescales by a threshold length of time such as those collective behaviours not lasting, for example, more than an hour, in this review we instead propose that long timescales are important for collective behaviour when there is a change in the biology of the individuals within the group; these can be relatively short-term changes in state (such as in knowledge or hunger), changes associated with ontogeny, or changes between generations over evolutionary timescales.

Previous reviews on collective behaviour have begun to focus on some aspects of change at temporal scales longer than those typically studied in collective behaviour. Biro *et al*. [9] discussed the importance of learning in collective behaviour, considering how being part of a group allows individuals to obtain information, how learning about past individual and collective performance can allow individuals to adapt their behaviour in future collective actions, and hence how learning can feed back into collective performance. Importantly for our special issue, Biro *et al*. [9] made the point that collective behaviour in animals is distinguished from collective behaviour in physical or chemical systems because of the potential for change over time in the individual units that make up the collective (i.e. the individual animals). Bengston & Jandt [26] explored how consistent inter-individual nbsp;behavioural variation between groups within the same population (i.e. ‘collective personality’) arises, particularly in social insects. They identified a multitude of factors that, over time, accumulate to lead to consistent differentiation between groups. This includes early experience, the age of the colony and its constituent members, and variation in the individuals that make up the colonies that results in different developmental trajectories.

Here in this introduction to the special issue ‘Collective animal behaviour through time’, we aim to provide a much broader (but correspondingly, much less in depth) view of the timescales relevant in collective behaviour, focused on empirical studies and structured to take the reader through the multiple timescales that collective behaviour can be studied across.

## 2. Aims of the themed issue

Collective behaviour is a truly inter-disciplinary field. It has relied on the quantitative approaches of physicists, computer scientists and mathematicians to investigate the mechanisms that underlie collective behaviour, and rigorous experimental approaches from behavioural biologists to provide empirical tests for these mechanisms and to understand its adaptive functions. Now the time is ripe to extend the study of collective behaviour further to include much longer timescales. This special issue is intended to encourage cross-pollination of disciplines by offering a unique opportunity for readers from traditionally disparate perspectives to be exposed to unfamiliar ideas that they can incorporate into their own work or use to build productive collaborations. Our goal is to spark a new wave of inter-disciplinary research that reinvigorates the field of collective animal behaviour. In addition to fostering new collaborations built on conceptual common ground, we aim to facilitate further research by combining methods from the study of collective behaviour, development and evolution, where there is great potential in inter-disciplinary approaches. Understanding changes in collective behaviour over long timescales also offers insight into how responsive collective behaviours can be to external influences. This is particularly timely as there is intense and growing research in how collective behaviour is affected by human-induced environmental change [27–29]. A comprehensive understanding of the different timescales over which collective behaviour can change has the potential to help inform which species that rely on collective behaviour for survival are vulnerable to environmental change, and if they are adaptable, how rapidly.

## 3. Rapid timescales (typically less than 1 second and seconds)

While we argue that the long timescales of development and evolution have been neglected in the study of collective behaviour, the existing literature in this field on shorter timescales still shows considerable variation in timescales, depending on the collective behaviour and the species of interest. Even when studying the same species and behaviour, studies can focus on the microscale interactions between individuals (e.g. [30,31]) or the macroscale group-level behaviour and outcomes of these interactions [32,33]. While some studies have used data at static instances in time, particularly analysing the spatial arrangement of individuals within groups [34–36], the short timescales in which inter-individual interactions occur have been given much more research attention, which typically happen over the timescale of seconds.

An intensely researched question within collective behaviour is how individuals interact within moving groups to achieve collective motion while maintaining group cohesion. Fish shoals have become a model system for examining these behaviours and broadly support models of collective motion [13,37] that ‘interaction rules’ within groups consist of prioritizing repulsion when neighbours are too close to avoid collision, and otherwise attracting to, and aligning direction of travel with, near neighbours. These interactions are determined by individuals rapidly adjusting their speed and direction of motion as the group moves [30,38]. In bird flocks, evidence suggests that collective movement is regulated by individuals adjusting their direction of movement rather than their speed, owing to the increased cost of adjusting speed during flight [39].

During collective motion, individual responses to information from near neighbours can occur very rapidly, in less than a second [7,40,41]. As changes in the direction of movement of the whole group require repeated interactions between individuals, this collective behaviour occurs at a longer timescale, such as seconds in fish shoals and bird flocks [42,43]. These changes in a group's direction can be the result of information transfer within the group [44,45], where the change in direction is owing to some individuals responding to a stimulus, such as a predator or food cue [46–49], and their response being copied by their near neighbours. Information can transfer within a group more rapidly than the approach of a predator [50], suggesting that some individuals in the group will be able to initiate anti-predator behaviour at a further distance from the predator than if they relied only on their own, i.e. private, information. The copying of a neighbour's behaviour can create waves of altered behaviour moving across the group, which can not only transfer information, but deter predators from attacking the group [51]. Experimental work has suggested that explicit consideration of sensory cues greatly improves the ability to predict information flow in fish shoals in contrast with classic models of collective motion [52], which are now being explored in more biologically realistic models of collective movement [53,54].

The existing literature on fish shoals and bird flocks suggests that the most widespread proximate mechanism for individuals responding to their near neighbours is copying the speed and direction of those near neighbours. This is a passive mechanism that does not rely on active signals or any other mechanism that has probably evolved specifically for this purpose (although see Jägers *et al*. [55] for an example of active signalling in fish shoaling). By contrast, there are a variety of mechanisms that ants and bees use to transmit information to one another. Some are also based on speed and direction, such as the streaking behaviour of scouts in bee swarms, where the scouts with knowledge of a new nest-site fly with an elevated velocity through the moving swarm in the direction of that site [56,57]. As in fish and bird collective movement, this is believed to be mediated primarily through vision [58].

Probably reflecting the importance of collective behaviour in social insects, the mechanisms for inter-individual interactions in these species are, however, more diverse and specialized than the passive mechanism in fish and bird groups. These mechanisms vary in spatial as well as temporal scale. At the small, local scale, the decision to engage in foraging activities in harvester ants (*Pogonomyrmex barbatus*) is mediated by direct antennal contact between two individuals [59], and traffic flows in ants are regulated by collisions between pairs of ants moving in different directions [60]. By contrast, the deposition of pheromones in ants does not require direct contact between individuals and allows influence of multiple other colony members, e.g. to recruit others to food [61,62]. The iconic and well-researched waggle dance of bees [63,64] is another important example of a specialized form of inter-individual interaction underpinning collective behaviour where information is made available to multiple other individuals simultaneously, although in this case the transmission of information is direct rather than indirect as is the case with pheromones. The variety in inter-individual interactions within social insects that underpin collective behaviour is reflected in the variable timescales that these interactions take place over; waggle dances can vary in duration up to multiple seconds [65], and while laying a pheromone takes less than a second [66], the pheromone can persist for minutes, making that information available to other colony members for an extended period of time [67]. While inter-individual interactions in social insects do often occur more slowly than the speed and directional changes in fish shoals and bird flocks, they can encode a greater range of information, such as the direction and distance of a food source [63–65].

## 4. Minutes, hours and days

While inter-individual interactions can be very rapid, these interactions give rise to group-level collective properties that change over longer timescales. Evidence exists that the density of groups (i.e. the spacing between individuals) can oscillate over time without any external influence, shown in both fish shoals [68] and sheep herds [19]. Changes in such collective state variables can arise owing to conflicts in the preferred state [46]. The timescale in which collective properties change can be, however, dependent on group size. For example, the direction of travel of locust swarms switches over time without any external influence, but the rate of switching reduces as the number of individuals' increases [33], which was later demonstrated to be driven by cannibalistic interactions between the locusts [31]. Despite the differences between locusts and fish in their morphological, sensory and cognitive traits, and their functional reasons for grouping, this dependence on group size has also been demonstrated in fish shoals [32]. In golden shiners *Notemigonus crysoleucas*, the degree of group polarization (fish being aligned and swimming in the same direction) is more stable over time at larger group sizes [32].

While two individuals in a group can exchange information very rapidly, group-level decisions are the outcome of many such decisions that occur through self-organized and decentralized processes, and thus such decisions will occur at longer timescales than individual interactions. Collective decision making has been studied most extensively in social insects, primarily in the context of foraging and relocating to a new nest site. This work has shown how consensus for a collective decision, for example towards a food source or nest site, requires time to build. A prominent example is how foraging trails form in ants that lay pheromones while foraging [61,62,66,69]. Here, the deposition of pheromones by foragers returning from food sources recruits subsequent foragers to the food source, who deposit additional pheromone which attracts further foragers to the source. This positive feedback gradually builds the foraging trail and can lead to the disproportionate use of one food source when multiple food sources of equal quality are available (known as symmetry breaking [69]). Quorum decision making is another well-researched example of how collective decisions take longer to occur than the decision making of the individuals contributing to that collective outcome [70]. Here, when the number of other individuals in the group (or the proportion of the group) that has chosen an option reaches a threshold, the probability that undecided individuals copy that choice increases dramatically, again triggering a positive feedback favouring that choice. In the ant *Temnothorax albipennis*, there is evidence that the number of encounters per unit time (i.e. the encounter rate) between individuals is the proximate mechanism that allows individuals to determine whether a quorum threshold has been met for a particular choice [71], and similarly in harvester ants, the decision to leave the nest to forage is influenced by the rate of encounters with individuals returning to the nest after foraging [59]. The accumulation of individual-level information over an extended period of time further emphasizes the importance of time in collective behaviour.

The pooling of individual-level information can provide animal groups with a greater cognitive ability to make decisions than the individuals making up the group [72–74]. However, what is evident in the collective decision making of some social insects is that group-level decisions can involve several distinct phases of different collective behaviours, so that the group decision has to take longer than any one of the phases. For example, in *Temnothorax rugatulus*, nest-site selection begins with scouts discovering potential sites and recruiting other members of the colony using tandem running [75], where informed scouts guide others to the potential site. Once a quorum threshold is met for a site, the behaviour switches to transport, where informed individuals physically carry others to the site [24]. Emigration to a new nest site in bees is also clearly divided into multiple phases [25,76], again beginning with scouts initially discovering and assessing potential sites. These scouts recruit others to sites using waggle dances at the colony. Once a quorum threshold of scouts visiting a particular site is met, scouts begin a piping signal at the nest to indicate that the colony should prepare to fly to the chosen site [77]. During flight to the new site, the scouts then streak through the swarm in the direction of the site [56,57]. Thus, the collective action of emigration to a new nest site can be a composite of different kinds of collective behaviour, occurring in a sequence that maximizes decision making efficiency. Additionally, there is evidence in both bees [76] and ants [78] that the timescale over which collective decisions are made can be variable to optimize the trade-off between speed and accuracy, which is achieved by adjusting the quorum threshold used to make a decision.

At longer temporal scales than a single collective action, a number of studies have investigated animal groups repeatedly performing a collective action. How the learning of individuals interacts with collective behaviour is covered in detail in Biro *et al*.'s [9] review, and studies in this area typically repeatedly test the same individuals in a collective task. Such repeated testing has shown important effects on diverse aspects of collective behaviour, including nest-site selection in ants and bees [79,80], homing in pigeons [81,82], and collective motion [83,84] and decision making [85,86] in fishes. Learning is one way that an individual's state can influence collective behaviour, as it involves an increase in an individual's state of knowledge. Changes in individuals' energetic state (i.e. nutritional or metabolic state) over time can also affect collective behaviour. A number of empirical studies have shown hungrier individuals take positions at the front of moving fish shoals, where feeding rates are higher [87,88]. Furthermore, as individuals at the front of shoals feed and incur metabolic costs of digestion over time, reducing their aerobic capacity for swimming, they move towards the rear of the shoal [89]. With increasing hunger, fishes have greater inter-individual distances and are more likely to be isolated [90], which may explain reduced collective behaviour over repeated tests in addition to acclimatization to the environment [85]. The importance of nutritional and metabolic state has also been demonstrated in pigeons (*Columba livia*) during long-duration flights, where over time the flock becomes more widely dispersed. This can be fatigue; birds tire over time and are less able to respond to their near neighbours to maintain group cohesion [91].

Differences in state, such as in knowledge or energetics, can also be mechanisms that drive differentiation between individuals within groups to adopt leader or follower roles. The spatial sorting in moving groups where hungrier individuals are found at the front suggests that they may have more influence on, and hence lead, the direction and speed of movement [40,41]. In the model of Rands *et al*. [92], leaders and followers emerged from pairs of individuals foraging from a refuge owing to diverging energetic states, even though the two individuals were initially identical. Although it is difficult to minimize inter-individual variation in empirical studies, there is evidence that over repeated trials where the environment becomes more familiar, influence over group movements becomes less egalitarian and instead led by a minority of the group [86]. Even with extensive evidence that a range of (relatively fixed) individual traits determine which individuals lead in animal groups [93], social feedback between individuals during collective decision making can magnify these differences over time, reinforcing leader and follower roles [94]. Such social feedback can also be important in determining which workers perform which tasks in social insect colonies, i.e. the division of labour [95–97].

Many of the processes discussed in this section can contribute to differences between groups in their behaviour where these differences are consistent over time. This collective personality variation is, by definition, stable over at least multiple days [26,98,99]. Consistent variation between groups can be driven by differences in the composition of the group [26], and/or be magnified over time as a result of social feedback or state-dependent changes in individuals [83]. Thus, consistency in collective behaviour over time, as well as variation over time, is important in understanding the diversity we observe in collective behaviour.

Here we have highlighted how considerable research effort has been spent to understand how collective behaviours occur as a result of individual decisions and the transfer of information. Longer timescales are involved when a collective action involves multiple phases, or repeated collective behaviours over time, resulting in changes in knowledge or energetic state. We now focus on expanding this time view even further to incorporate aspects of developmental and evolutionary change in individual behaviour to better understand how collective behaviour develops throughout the lifetime and evolves across generations.

## 5. Development: changes over the lifetime

The study of ontogeny investigates how an individual's traits arise and change over their lifetime, and the drivers that influence these changes. Bengston & Jandt [26] review the developmental changes that colonies of social insects undergo, such as the tendency for colonies to become more aggressive as they increase in age and size [100]. This group-level approach is most relevant to groups with stable membership such as eusocial insect colonies. Here, we discuss the importance of the ontogeny of individuals in collective behaviour, which has wider taxonomic relevance as it includes animals that live in more loosely formed groups. As already mentioned, variation between individuals in their traits can be important in collective behaviour, and changes over developmental time as an animal reaches and passes maturity are associated with altered physiology, morphology, sensory systems and cognition, all of which can impact collective behaviour [93]. However, such age-related changes have been relatively neglected in the field of collective behaviour compared to individual-level traits which vary over shorter timescales (such as changes in knowledge or energetic state), or those traits which are fixed in the lifetime of (most) animals, such as sex.

Shoaling behaviour during the early life of fishes is one aspect of the development of collective behaviour that has attracted some research attention. This research shows that fish larvae do not demonstrate collective behaviour from birth, rather it develops as the individual grows. In Spanish mackerel *Scomberomorus niphonius*, for example, close neighbour distances and coordinated schools of fish swimming in the same direction (i.e. demonstrating polarization) develop between 17 and 19 days post hatching, without further change from day 19 to 23 [101]. By contrast, Nakayama *et al*. [102] showed that in chub mackerel (*Scomber japonicus*), there is a gradual increase in individuals' polarization with neighbours from 10 days post hatching. However, they also showed that the social cue of a fright stimulus from conspecifics does not induce a response until two weeks later in development, and linked these developmental changes in collective behaviour to the change in the volume of the optic tectum over this time period [102]. The importance of the development of the visual system in early life was also demonstrated in striped jack *Pseudocaranx dentex*, where larger (and hence older) fish were able to school at lower light levels than smaller (younger) fish [103]. Providing a sensory basis for developmental changes in collective motion illustrates the importance of determining the proximate mechanisms underlying variation observed in collective behaviour, a point discussed in detail by Jolles *et al*. [93].

Of particular promise in studying collective behaviour from a developmental perspective is the zebrafish, *Danio rerio*. This species shoals under both natural [104] and laboratory conditions [105], and is a major vertebrate model organism in developmental biology and genetics [106]. A handful of studies have shown the potential of zebrafish in expanding our understanding of the early ontogeny of collective behaviour. As with other fishes, there was no evidence of collective behaviour in the very first days of life in zebrafish, but rather they show a gradual increase in cohesion (after controlling for body size) over their early lives [107,108]. Studies have linked the ontogeny of collective behaviour in this species to dopamine levels in the brain [109]. Altogether, this suggests that zebrafish show similar patterns of development in collective behaviour as other species and may be a very promising study system to investigate the proximate mechanisms generating change in individual and hence collective behaviour throughout early ontogeny.

The importance of age in leadership during group decision making is another case where the ontogeny of individuals has been shown to have importance in collective behaviour. Studies on this question are more taxonomically diverse than those examining the early onset of collective motion, which have been focused on fishes as discussed above, and also emphasize the importance of older, usually more experienced individuals within groups. It has been shown that older individuals are often at the front of moving groups, where they typically have more influence on the direction of the group's movement, for example this is found in greater white-fronted geese (*Anser albifrons*) [110], killer whales (*Orcinus orca*) [111] and muskoxen (*Ovibos moschatus*) [112]. Similarly, differentiation into leader and follower roles can be more stable when age is more variable within the group [113]. Importantly, the presence of older, more experienced individuals in the group has been shown to have benefits for those following, including in migratory performance in whooping cranes (*Grus americana*) [114] and predator avoidance in African elephants (*Loxodonta africana*) [115].

Where the importance of individuals' ages in collective behaviour has been most intensely researched in is the division of labour within social insect colonies [116]. The workers in colonies of many social insects shift from tasks within the nest when young, such as caring for brood, to riskier tasks such as foraging as they age, a phenomenon known as age (or temporal) polyethism [117]. Age polyethism is typically more pronounced in species with more advanced eusociality in contrast with those that are primitively eusocial [118], suggesting that it contributes to the success of eusociality, a highly advanced collective behaviour. The social environment can, however, influence the age that individuals change tasks in some species [119]. For example, a depletion of foragers induces younger individuals to start foraging earlier, as demonstrated in honeybees *A. mellifera* where this change was also associated with corresponding changes in workers’ juvenile hormone [97]. There is additional evidence for changes in gene expression [120], lipid reserves [121], musculature [122] and brain morphology [123] associated with changes in the tasks that individuals carry out [119]. Interestingly, where it is possible to separate age and experience, there is evidence that experience with different tasks, rather than age *per se*, is the driving factor for these changes [121,123]. Thus, while age and other factors such as the social environment (the number of other individuals engaged in different tasks) might trigger the change to a different task, it is experience with the task that leads to these individual-level changes. Flexibility in the age that individuals engage in different tasks provides plasticity for the colony in response to variable conditions, alongside the advantage of efficient use of workers associated with age polyethism.

While there has been considerable research investigating how physiology, sensory systems and behaviour develop over an individual's early life, less research has focused on how collective behaviour is thus influenced by these developmental changes. As individuals develop they will experience changes in their ecology becoming more or less vulnerable to predators, begin seeking mates, or inhabiting new environments, and it is currently unclear how patterns of collective behaviour shift in response to these changing pressures, and how collective behaviour can help mitigate such demands too. Here we see fruitful research avenues that can take a more holistic, long-term view of collective behaviour over an animal's lifetime.

## 6. Changes over evolutionary time

Of the timescales studied in biology, arguably the least well advanced in the study of collective behaviour is the longest, that of change over generations. This is somewhat surprising as the functional explanation (i.e. the adaptive value of a behaviour in Tinbergen's four questions [8]) for living in groups has been researched for many decades [1–3], and more specifically, the benefits of a diverse range of collective behaviours have been demonstrated [22,24,36,46–48,65,74]. Here, we review approaches that have been used to study the evolutionary history of collective behaviours.

A core tool in the evolutionary biologist's toolbox is the comparative approach, i.e. comparing the traits of different species. Together with knowledge of the evolutionary tree of how these species are related, it can reveal, for example, whether similar traits across species are conserved from a common ancestor, or have evolved independently (convergent evolution). The differentiation into queen and worker castes in social insect colonies is one such example of convergent evolution, where this has multiple origins in the evolutionary past of bees, ants and wasps [124]. Comparative studies can also test whether the presence of multiple traits is correlated across species, indicating coevolution of these traits. For example, warning coloration (aposematism) is associated with the transition from group to solitary living in caterpillars [125], eusociality is associated with a decrease in the size of central processing brain regions in wasps [126] and long-distance migration is associated with larger flocks while in flight in birds [127].

These examples of studies that explore the evolutionary history of social behaviour use measures that are relatively easy to characterize, such as the degree of eusociality or group size. Often these data are pre-existing in the scientific literature or in databases such as FishBase [128], facilitating the comparison of many species which improves test power as well as allowing for phylogeny (the evolutionary relatedness between species) to be included in the analysis. However, many of the collective behaviours that researchers are most interested in, usually those occurring on timescales from seconds to days as discussed previously, take considerable effort for researchers to measure and quantify. This is probably explains why comparative studies of collective behaviour are usually limited to very few species in comparison to typical comparative studies. In an early example from fish shoaling, Partridge *et al*. [34] carried out detailed measurements under laboratory conditions of the shoal shape and structure of three species of fishes which varied in their schooling tendency. In marine field conditions, Burford *et al*. [129] compared inter-individual distances, alignment between neighbours, and the speed of alignment with neighbours between two taxonomically very different species, California market squid (*Doryteuthis opalescens*) and Pacific sardine (*Sardinops sagax*). Bisazza *et al*. [130] measured shoal cohesion and behavioural lateralization in 16 species of fishes under laboratory conditions, with shoal cohesion quantified from the number of fishes located in the same sections of a grid. Understandably given the trade-offs generated by the constraints of empirical work, the trend here is that where collective behaviour is studied in natural conditions and/or in greater detail, fewer species can be studied.

A similar trend exists in comparative studies of collective behaviour in social insects. Latty & Beekman [131] compared five ant species in their use of dynamically changing foraging resources. Hölldobler *et al*. [132] found similarity in the chemical composition of pheromones used for recruiting colony members to food in four species of harvester ant. Four species of ant were also compared in Creery's [133] study of collective transport. Three species were compared in Muscedere & Traniello's [134] comparative study of the relationship between the relative sizes of different brain regions and division of labour in *Pheidole* ants, and Beekman *et al*.'s [64] comparative study of the waggle dance involved three species of *Apis*. Czaczkes *et al*. [135] compared the mechanism of recruiting other ants to cooperatively retrieve large food items in the longhorn crazy ant *Paratrechina longicornis* to two unrelated species of ant. Such studies are essential to understand the extent of generality versus specialization of different collective behaviours, as well as their evolutionary history. The current challenge is to develop protocols that can quantify collective behaviour with a higher throughput, facilitating comparison of far more species.

Rather than compare species, a similar approach is to make comparisons across populations of the same species, where the populations have been isolated for a sufficient length of time that they have adapted to local environmental conditions. Such comparisons are most reliable when there are multiple, independent populations evolving across a range of the environmental parameter of interest. A classic example is the guppy in Trinidad (*Poecilia reticulata*), which lives in geographically isolated rivers in the Northern Range mountains. Waterfalls and rapids limit the dispersal of predator fish species upstream, so that in many of these rivers, the downstream stretches are high-predation risk habitats, in contrast with the low-predation habitats upstream [136]. Early studies revealed that guppies from sites with high-predation pressure form larger shoals [137]. More recently, comparing guppies from rivers varying in predation risk has demonstrated differences in their fine-scale inter-individual interactions during collective motion [138], and in collective decision making when tested both *in situ* in the field [139] and under standardized conditions [140]. Further evidence that collective behaviour adapts to local environmental conditions within species comes from the Mexican tetra (*Astyanax mexicanus*), where ancestral populations live in rivers exposed to light (surface populations) and show attraction and alignment to their neighbours, while multiple populations of this species have independently adapted to live in dark caves and show avoidance of conspecifics [141].

While research comparing across populations of the same species is studying evolutionary change that has occurred over a shorter timescale than comparative studies across species, an even shorter timescale is involved in artificial selection experiments which can involve only a few generations [142]. Here, researchers choose individuals that will breed to create the next generation based on a trait (or traits) of interest, thus the selection process is artificial. This has the potential to reveal the speed at which traits can evolve, and how other traits covary with selection on the trait being directly selected for. Applying this to research on collective behaviour is still in its infancy but has been used in some species of fishes that have generation times typically shorter than in birds or mammals (as well as being considerably easier to maintain under laboratory conditions), and do not have the complication of eusocial insects where selection acts primarily on the colony, rather than the individual. Kotrschal *et al*. [143] bred guppies (*P. reticulata*) for increased alignment with neighbours (i.e. polarization). Within three generations compared to control (non-selected) fish, the artificially selected fish increased both polarization and group cohesion, corresponding to stronger responses in alignment and attraction to neighbours. Selection for polarization was not strongly associated with changes in cognitive traits of the individual guppies [144], contributing to the ongoing debate regarding the link between group living and individual-level cognitive abilities.

Artificial selection on non-social traits has documented impacts on collective behaviour. Goldbelly topminnows (*Girardinus falcatus*) artificially selected for behavioural lateralization were more cohesive and more aligned in the direction of travel with neighbours than individuals selected for low lateralization [145]. In zebrafish (*D. rerio*), artificial selection has been used to recreate size-selective mortality, which is common in fishes from their natural predators and fishing by humans. Fishes selected for larger body sizes (mimicking harvesting of smaller individuals, typical of natural predation) formed more cohesive shoals than controls, while those selected for small body size (mimicking harvesting of larger individuals, typical of human fishing) formed less cohesive shoals [146]. Studies of these fishes have also shown how the change over ontogeny in group cohesion [147] and collective foraging under predation risk [148] can be affected by size-selected mortality, illustrating that there can be interactions across developmental and evolutionary timescales in collective behaviour.

A major consideration in the evolutionary change of collective behaviour is the heritability of this trait. The tendency to be social has been shown to be heritable across a range of taxa (reviewed by Gartland *et al*. [149]), and there is evidence that some collective behaviours are heritable. Seghers' ([137], also see [150]) early study of local adaptation to predation risk in the guppy (*P. reticulata*) included raising guppies from different populations under the same conditions (i.e. in a common garden experiment) without predators for 3–4 generations, and showed that differences in shoal cohesion between fish from high- and low-predation environments persisted over generations, revealing a heritable component of the shoaling differences. Heritability in attraction to and alignment with neighbours has also been demonstrated in three-spined sticklebacks (*Gasterosteus aculeatus*) by comparing fish from marine (strongly schooling) and freshwater benthic (weakly schooling) populations [151]. There is also evidence from the pharaoh ant (*Monomorium pharaonis*) that collective foraging, aggression and exploration are heritable, as well as being correlated with one another [152].

Corresponding to the heritability of collective behaviour, a number of studies have explored its genetic basis. In social insects, the heritability of, and genes underlying, the division of labour between workers has been reviewed by Smith *et al*. [153]. For example, the expression of the *foraging* gene plays a major role in regulating switching between different behavioural tasks in workers in a range of social insects [154]. Although a small number of strains of zebrafish (*D. rerio*) have been compared and shown to vary in their collective motion [109,155,156], Tang *et al*.'s [157] study used a comparison of wild-type fish with 90 mutant lines created with CRISPR-Cas9 to demonstrate the genetic basis for differences in inter-individual spacing and polarization in shoals. The *Ectodysplasin* (*Eda*) gene has been identified to play a major role in the differences between marine and freshwater benthic populations of three-spined sticklebacks (*G. aculeatus*) in their collective motion [158]. By breeding marine and freshwater benthic three-spined sticklebacks to create F_2_ hybrids, Greenwood *et al*. [159] found that the strength of attraction to neighbours and alignment of orientation to neighbours are uncorrelated, and are associated with different genomic regions. Kowalko *et al*. [160] also used crosses of different populations, in this case surface and cave populations of the Mexican tetra (*A. mexicanus*), to demonstrate that schooling tendency is a dominant trait, and loss of schooling in cave populations over evolutionary time is associated with the loss of vision. Interestingly, while Kowalko *et al*. [160] found only a small role for changes in the lateral line sensory system in explaining the differences in schooling behaviour in the Mexican tetra, Greenwood *et al*. [159] identified a genetic link between schooling and variation in the lateral line in three-spined sticklebacks. Overall, these studies demonstrate the range of methods that can be used to determine the genetic basis of collective behaviour, yet the challenge now is to test a greater range of species to determine the generality of the findings so far.

## 7. Concluding remarks

Our special issue brings together leaders in collective animal behaviour and biologists with expertise in studying the development and evolution of behavioural traits. The primary objective is for researchers to recognize the untapped potential in studying collective behaviours from novel perspectives. In this introduction to the special issue, we provide an overview of the current state of our understanding of collective behaviour over highly variable timescales that have been applied to the study of these incredible animal behaviours.

For brevity, we have focused our review here on types of collective behaviour that have been extensively researched, such as nest-site selection in social insects and shoaling in fishes. Other collective behaviours exist and broadening studies to include other behaviours and systems could provide fruitful opportunities for comparative studies and assessing the generalities of our findings. For example, the collective architectures of social insects can be critical to a colony's survival; in this issue, Muratore & Garnier [161] discuss how these structures can be studied using a developmental biology approach. Rathore *et al*. [162] make the argument that lekking, which occurs when multiple males congregate to perform mating displays, might be best viewed through the lens of collective behaviour, whereby the emergent properties of the lek can be understood as the result of small, local-scale individual-level interactions. Also, while much of collective behaviour research has focused on fishes, birds and eusocial insects, McLellan & Montgomery [163] highlight how caterpillars (i.e. Lepidopteran larvae) exhibit many of the same characteristics we value in these other animal taxa and may be especially tractable systems for investigating the development and mechanistic underpinnings of collective behaviours.

Considering a wider scope than our review here covers, there are also a number of broader contexts involving collective behaviour where variable timescales will be important. The interactions between groups can themselves give rise to interesting dynamics. In groups of stable membership, this can result in conflict or cooperation between the groups (the focus of Rodrigues *et al*.'s [164] article in this special issue), while in free-entry groups, i.e. those where group members are free to join and leave, fission–fusion dynamics can be important [165]. Collective behaviour can also be important in responding to dynamically varying resources [131,166] and environmental conditions [167,168]. An additional layer of complexity exists when considering the dynamics of animal groups interacting with other biological agents under selection, such as predators and (specifically, horizontally transmitted) diseases [48,51,169–171], where the interactions can again take place over multiple timescales. We also do not consider in our introduction review the collective behaviour of mixed-species groups [172], the topic of Lukas *et al*.'s [173] contribution to the special issue. Mixed-species grouping will typically involve substantial within-group inter-individual variation, as different species will have different response times to stimuli, developmental trajectories, and rates of adaptation.

While there is great potential in studying collective behaviour over developmental and evolutionary timescales, there are substantial challenges comparing behaviours across time and species. The contributions to the special issue by Sridhar *et al*. [174] and Ogino *et al*. [175] both analyse metrics of collective behaviour at a range of timescales, and demonstrate that the conclusions drawn are dependent on the timescale chosen for analysis. Romero-Ferrero *et al*. [176] develop new analytical tools to track how information can flow among members of the same group. Sumner *et al*.'s [177] article in this special issue investigates how analysing genes and genomes can reveal whether highly advanced collective behaviour (in this case, colonies of social insects that can be defined as superorganisms) evolved gradually in small increments or in a nonlinear, stepwise trajectory. This approach is possible, in part, because superorganismality is well defined and relatively easily quantified to exist, or not exist, in different species. However, like many behavioural traits, other collective behaviours are frequently context specific, making comparisons across species difficult [130]. For example, should we quantify and compare shoaling across fish species under identical laboratory conditions, even though some species may be more stressed under these conditions than others? Even where observational field studies are possible, variation in the habitats occupied by different species could explain differences observed in collective behaviour, rather than be representative of direct differences between species. A related issue for cross-species comparisons is in using the same approach to quantifying collective behaviour. Although a large array of metrics have been developed to quantify collective behaviour, measures such as inter-individual distances and polarization are frequently used, and Papadopoulou *et al*.'s [178] article in this special issue demonstrates how these metrics can be used to directly compare the collective behaviour of taxonomically and ecologically diverse species.

Other challenges are common to studies of collective behaviour in general but are likely to be particularly relevant to developmental studies. A major theme in the field of collective behaviour is that the group-level ‘macroscopic’ properties are often difficult to predict from the ‘microscropic’ inter-individual interactions that drive the group-level behaviour. These emergent properties suggest that developmental changes at the individual level may not correspond to changes at the collective level. For example, Rolland *et al*.'s [179] study of ageing in the slime mould *Physarum polycephalum* could be extended further by investigating how developmental changes can be explained by the components contributing to decentralized decision making in these single-celled organisms. Siracusa *et al*. [180] examine how ageing in free-living rhesus macaques *Macaca mulatta* can impact individual- and group-level social properties. Here, older individuals show changes in their social behaviour, but these individual-level changes may not scale up to affecting whole networks. This could in part be because unlike most traits, the collective behaviour an individual expresses is reliant on other members of the group, thus separating out an individual's behaviour from the behaviour of others can raise difficulties. Even under controlled experimental conditions, the behaviour of a focal individual can be dependent on the behaviour of others in the group [181], and those groupmates can vary considerably given the degree of inter-individual variation that has been demonstrated to be important in collective behaviour [93]. In some species without groups of stable membership, short-term studies can overcome this by testing the same individual with different groupmates. Wice & Saltz [182] exploit the powerful fruit fly system to generate replicated social groups to better investigate how social interactions among individuals influence entire social networks. Finding both direct and indirect genetic effects on an individual's social position within the network opens up the possibility for the evolution of the entire social environment. Over longer timescales, the developmental trajectory of an individual's behaviour may be shaped by the collective behaviour (as part of the social environment) it experienced in the past. Learning is a prime example of this and Collet *et al*. [183] provide a framework for conceptualizing how collective learning might occur in groups. In particular, they propose a number of different mechanisms through which individuals can learn and adjust their behaviour to improve collective performance, providing testable predictions for each.

We hope our special issue will drive forward new avenues of research using both theoretical and empirical approaches to investigate why and how we would expect collective behaviour to change over time. We aim to inspire readers to consider novel directions for their research, either by integrating developmental and evolutionary perspectives into their existing research for those already working on collective behaviour, or by including collective behaviour as an understudied trait of interest for those already working in developmental or evolutionary biology.

## Data Availability

This article has no additional data.
